# Co-expression of P173S Mutant Rice *EPSPS* and *igrA* Genes Results in Higher Glyphosate Tolerance in Transgenic Rice

**DOI:** 10.3389/fpls.2018.00144

**Published:** 2018-02-13

**Authors:** Dhirendra Fartyal, Aakrati Agarwal, Donald James, Bhabesh Borphukan, Babu Ram, Vijay Sheri, Renu Yadav, Mrinalini Manna, Panditi Varakumar, V. Mohan M. Achary, Malireddy K. Reddy

**Affiliations:** ^1^Crop Improvement Group, International Centre for Genetic Engineering and Biotechnology, New Delhi, India; ^2^Department of Biotechnology, Uttarakhand Technical University, Dehradun, India; ^3^Plant Molecular Biology Lab, Department of Botany, University of Delhi, New Delhi, India

**Keywords:** weed control, herbicide tolerance, glyphosate, EPSP synthase, igrA

## Abstract

Weeds and their devastating effects have been a great threat since the start of agriculture. They compete with crop plants in the field and negatively influence the crop yield quality and quantity along with survival of the plants. Glyphosate is an important broad-spectrum systemic herbicide which has been widely used to combat various weed problems since last two decades. It is very effective even at low concentrations, and possesses low environmental toxicity and soil residual activity. However, the residual concentration of glyphosate inside the plant has been of major concern as it severely affects the important metabolic pathways, and results in poor plant growth and grain yield. In this study, we compared the glyphosate tolerance efficiency of two different transgenic groups over expressing proline/173/serine (P173S) rice *EPSPS* glyphosate tolerant mutant gene (*OsmEPSPS*) alone and in combination with the glyphosate detoxifying encoding *igrA* gene, recently characterized from *Pseudomonas*. The molecular analysis of all transgenic plant lines showed a stable integration of transgenes and their active expression in foliar tissues. The physiological analysis of glyphosate treated transgenic lines at seed germination and vegetative stages showed a significant difference in glyphosate tolerance between the two transgenic groups. The transgenic plants with *OsmEPSPS* and *igrA* genes, representing dual glyphosate tolerance mechanisms, showed an improved root-shoot growth, physiology, overall phenotype and higher level of glyphosate tolerance compared to the *OsmEPSPS* transgenic plants. This study highlights the advantage of *igrA* led detoxification mechanism as a crucial component of glyphosate tolerance strategy in combination with glyphosate tolerant *OsmEPSPS* gene, which offered a better option to tackle *in vivo* glyphosate accumulation and imparted more robust glyphosate tolerance in rice transgenic plants.

## Introduction

Weeds are the most serious biological constraints in agriculture, which have enormous impact on crop plants in terms of growth, development, and grain yield. They instantly highjack the whole agricultural field and aggressively compete with crop plants for nutrients, water, sunlight, and space. Weeds also act as an alternate host for major insects, pests, and diseases (Burnside and Wicks, 1969; Kraehmer and Baur, 2013). In addition, some harmful weeds release phyto-toxins in soil which negatively affect the growth of crop plants. The traditional weed control practices, including hand picking of the weeds, burning the weeds before sowing of new crops, ploughing, harrowing, flooding the whole field with water and crop rotation, are very efficient methods in small cultivation fields. However, it is quite difficult to manage weed infestation over large cultivation areas using these methods due to their labor intensive and time-consuming nature (Masthan et al., 1989; De Datta and Barker, 1997; Zhang, 2003). On the other hand, the chemical based weed control practices are highly effective and economical, and offers a better option for achieving higher productivity. So, developing genetically engineered crop plants for herbicide resistance and applying non-selective herbicides, to kill or suppress various kinds of weeds without affecting the fitness of crop plants, is considered as one of the most valuable tool in modern integrated weed management system.

Glyphosate is the leading post-emergent, non-selective, and broad-spectrum herbicide to control various kinds of annual and perennial weed populations. The systemic nature of herbicide kills all the plants indiscriminately by inhibiting 5-enolpyruvyl shikimate 3-phosphate synthase (EPSPS), an important plant enzyme responsible for biosynthesis of aromatic amino acids and other crucial plant metabolites (Duke and Powles, 2008, 2009). The first commercial success of glyphosate transgenics was reported in soybean in 1996 by Monsanto company. The intensive and continuous application of glyphosate and lack of diverse weed management practices imposed a high selection pressure on weed population which, unfortunately, resulted in development of resistant biotypes (Heap, 2017). The first glyphosate resistant rigid ryegrass (*Lolium rigidum* Gaudin) was reported in Australia in 1996 (Green and Owen, 2011).

The resistant weeds are frequently evolved by modifying amino acid composition on the substrate binding site of EPSPS under high glyphosate selection pressure. Among the evolved glyphosate tolerant amino acid substitution mutations, the substitution of proline at 106 to serine is frequently reported in many resistant biotypes (Supplementary Table 1 for references). In addition to EPSPS target site modifications, some other glyphosate resistance mechanisms have also been evolved in plants due to high copy number of *EPSPS*, reduced translocation of glyphosate to meristematic region and increased vacuolar sequestration, collectively known as non-target site modifications (Lorraine-Colwill et al., 2002; Gaines et al., 2010; Ge et al., 2010; for review, see Powles and Preston, 2006; Shaner, 2009; Powles and Yu, 2010; Sammons and Gaines, 2014). All these mechanisms endow a moderate-level of glyphosate resistance to plants. To effectively control these resistant weeds under field conditions, a higher dose of glyphosate application is required which is also breaching the threshold limits of transgenic crops for glyphosate tolerance.

The continuous and high dose application of glyphosate has theoretical disadvantages to plant as it is stable inside the plant cell, does not get metabolized and remains accumulated. Glyphosate is rapidly translocated to actively growing meristematic tissues and reproductive organs from various parts which in turn results in retarded plant growth and lower crop yield (Pline et al., 2002; Chen Y.C. et al., 2006; Liang et al., 2017). So, the existing glyphosate tolerant EPSP synthase technology is complemented with detoxification systems for effective removal of accumulated glyphosate residues which result in more robust tolerance along with proper plant growth and development (Dun et al., 2014; Guo et al., 2015). There are many glyphosate-metabolizing bacterial enzymes, e.g., *glyphosate N-acetyltransferase* (*GAT*), *glyphosate oxidoreductase* (*GOX*), *glycine oxidase* (*GO*) and *D-amino acid oxidase* (*DAAO*) which have been identified and successfully used in various crops for development of glyphosate tolerant transgenic plants. The *increased glyphosate resistance* (*igrA*) is another bacterial enzyme, reported in 1990 and still less explored, that metabolizes glyphosate into sarcosine and inorganic phosphate, and subsequently into formaldehyde and glycine (Fitzgibbon and Braymer, 1990).

In this study, we analyzed the effective contribution of glyphosate degrading encoding *igrA* gene in transgenic plants, in combination with P173S glyphosate tolerant rice *EPSPS* mutant (*OsmEPSPS*) gene, to impart glyphosate tolerance. We developed two groups of transgenic rice plants expressing either *OsmEPSPS* alone, referred as ‘S’ transgenic lines, or in combination with *igrA* gene, referred as ‘D’ transgenic lines. After comparing the glyphosate tolerance efficiencies of both groups by various expression and physiological experiments, it was concluded that the co-expression of both *OsmEPSPS* and *igrA* genes could be an effective strategy for developing more robust glyphosate tolerant transgenic rice plants since the strategy is a combination of two different glyphosate tolerance mechanisms.

## Materials and Methods

### *In Silico* Analysis of Plant EPSPS Protein Sequences

The EPSPS protein sequences from 10 different plant species, i.e., *Oryza sativa, Arabidopsis thaliana, Eleusine indica, Gossypium hirsutum, Nicotiana tabacum, Solanum lycopersicum, Sorghum halepense, Triticum aestivum, Lolium multiflorum*, and *Zea mays*, were obtained from NCBI database. The multiple sequences were analyzed using MEGA 6 to identify the conserved proline amino acid position in rice EPSPS which was mutated to serine to make the enzyme glyphosate tolerant.

### Introduction of P173S Amino Acid Substitution in Rice *EPSPS* Gene

**FIGURE 1:**
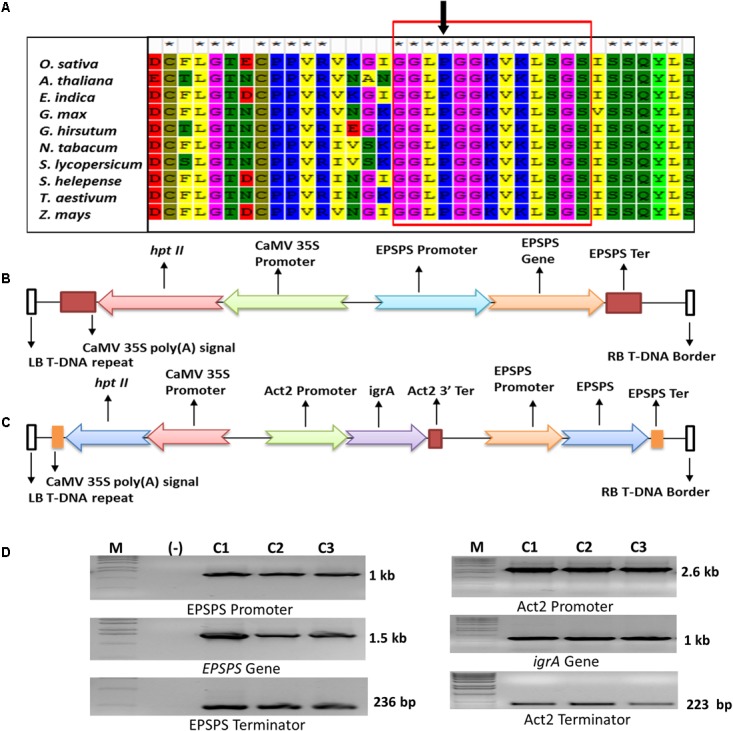
Sequence analysis of EPSPS proteins, diagrammatic representation and PCR confirmation of genes in plant expression cassettes. Multiple sequence alignment of EPSPS protein sequences from various plants species to identify the position of conserved Proline-106 amino acid. The arrow shows the position of conserved proline at 173 amino acid in rice EPSPS **(A)**. Schematic representation of plant expression cassette of ‘S’ and ‘D’ transgene constructs **(B,C)**. Confirmation of *OsmEPSPS* and *igrA* gene cassettes in entry vectors by PCR where M denotes 1kb ladder; (-) denoted negative control and C1, C2, C3 denote three individual bacterial colonies **(D)**.

The *in silico* analysis of various plant EPSPS polypeptide sequences revealed a conserved position of proline amino acid at position 173 (173 in rice and 182 in *Arabidopsis*), which is replaced with serine in several glyphosate resistant weeds (Supplementary Table 1). We selected this mutation point at 173 and replaced it with serine in rice *EPSPS* gene by PCR based mutagenesis technique to make it glyphosate tolerant. The full length 1,548 bp *EPSPS* gene was PCR-amplified using sequence specific primers from cDNA of indica rice variety Swarna in two fragments. The first fragment, containing 540 bp from 1 to 540 bp, was amplified by using primers EPSPS-AF and EPSPS-AR while the second fragment, containing 1,028 bp from 520 to 1,548 bp, amplified with the help of primers EPSPS-BF and EPSPS-BR with 20 bp long overlapping region (Supplementary Table 2). The fragments were PCR amplified using 150 ng of respective primers, 200 mM of dNTPs, 2.0 unit of *Taq* polymerase and 0.5 μl of DMSO in 50 μl reaction. The steps in PCR cycle were used as 94°C for 1 min, 55°C for 1 min, and 72°C for 1 min for 30 cycles with an initial denaturation at 94°C for 4 min and final extension at 72°C for 10 min. We replaced the native CCA codon (coding for proline at position 173) with AGC codon (Ser) by designing specific primers EPSPS-AR and EPSPS-BF overlapping primers and introduced a new *Hind*III restriction site which was absent in native *EPSPS* sequence (Supplementary Table 2). The full length mutated rice *EPSPS* gene sequence (*OsmEPSPS*), encoding P173S substitution, was generated by digesting the two fragments and ligating them back with the help of *Hind*III and T4 DNA ligase enzymes, respectively. The rice EPSPS promoter (1,036 bp) and rice EPSPS 3′ terminator (236 bp) regions were separately PCR amplified from rice genomic DNA using sequence specific primer pairs EPSPS-PF – EPSPS-PR and EPSPS-TF – EPSPS-TR, respectively (Supplementary Table 2). The rice full-length *OsmEPSPS* was cloned under regulatory sequences of rice native *EPSPS* as expression cassette (**Figure 1**) in Gateway^®^ compatible entry vector 1 (pL12R34-Amp). For making expression cassette with *igrA*, the rice codon optimized synthetic 1,058 bp *igrA* coding DNA sequence was cloned under rice actin 2 promoter (2,578 bp, amplified using Act2-PF and Act2-PR primers) and its 3′ terminator (223 bp, using primers Act2-TF and Act2-TR) in Gateway^®^ compatible entry vector 2 (pL34R12-Cm-ccdB) (Chen Q.J. et al., 2006).

### Preparation of Binary Vectors and Generation of Transgenic Plants

The *OsmEPSPS* expression cassette (*OsEPSPS-P*:*OsmEPSPS*:*OsEPSPS-T*) in entry vector 1 was finally stacked into plant transformation vector, pMDC99 (Curtis and Grossniklaus, 2003) using Gateway cloning method. The resultant recombinant *pMDC99-OsmEPSPS* vector was transferred to *Agrobacterium* EHA 105 strain, which was subsequently used for rice transformation to express *OsmEPSPS* alone to develop ‘S’ transgenic rice lines. For making two-gene expression cassette, we sequentially pyramided the expression cassettes of *OsmEPSPS* from entry vector 1 (pL12R34-Amp) and *igrA* expression cassette (*OsAct2-P*:*igrA*:*OsAct2-T*) from entry vector 2 (pL34R12-Cm-ccdB) into plant transformation vector pMDC99. The *Agrobacterium* EHA 105 colonies, harboring the recombinant *pMDC99-OmEPSPS-igrA* vector, were used for plant transformation to develop ‘D’ transgenic rice lines co-expressing both genes.

The indica rice cultivar Swarna was used for *Agrobacterium* mediated transformation. The embryogenic calli, developed from mature rice seeds, were infected and co-cultivated with *Agrobacterium* culture containing either recombinant *pMDC99-OsmEPSPS* or recombinant *pMDC99-OsmEPSPS-igrA* vector separately. The transformed calli were selected on 50 mg/l hygromycin with three rounds of selections and putative transgenic rice plants were generated from secondary calli after regeneration and rooting according to Chandrasekhar et al. (2014).

### Identification and Molecular Conformation of Transgenic Events

The putative transgenic plants were initially screened for the presence of *OsmEPSPS* with the help of PCR using sequence specific primers EPSPS-F and EPSPS-R (Supplementary Table 2). These primers were expected to amplify either 615 bp (we observed that most of the time PCR is biased to amplify this band in transgenic plants, however, native band is also amplified sometimes together) or 1,677 bp, or both DNA fragments in transgenic lines, and only 1,677 bp DNA fragment from non-transgenic rice plants. The 1,677 bp fragment was amplified from the native copy of rice *EPSPS* due to the presence of three introns (intron 1, 2, and 3). The *igrA* specific primers, igrA-F and igrA-R, were expected to amplify DNA fragment of 1,056 bp length from transgenic plants, and no amplification from wild type (*wt*) plants. The PCR was performed using 200 ng template genomic DNA from putative transgenic lines and *wt* plant separately. All the PCR conditions and components are same as mentioned above. The amplified DNA PCR products were resolved on 1% agarose gel followed by visualization on UV gel documentation system.

The genomic DNA was isolated following the protocol described by Dellaporta et al. (1983). For southern analyses, 10 μg of RNAse treated pure genomic DNA, from transgenic and *wt* plant lines, was separately digested with *Nco*I restriction enzyme. The restricted DNA fragments were separated on 0.8% agarose gel and subsequently transferred to positively charged nylon membrane using capillary movement method. The membrane was then sequentially hybridized with *EPSPS* DIG probe, amplified with EPSPS-F and EPSPS-R primers (to identify both ‘S’ and ‘D’ transgenic lines) and proceeded for final unique DNA signal detection. The hybridization, post hybridization washing conditions and detection of signals were performed according to manufacturer’s instruction (Roche Diagnostics, Germany). Following, probing with *EPSPS* gene, the same nylon membrane was reprobed with DIG labeled *igrA* probe, amplified with igrA-F and igrA-R primers, and further steps were followed as described earlier.

The genome walk technique was used to identify the integration site of transgene expression cassette in the genome of best transgenic event showing higher glyphosate tolerance following the method described by Reddy et al. (2002). At least 2 μg genomic DNA from best transgenic line was digested with *Bgl*II restriction enzyme and ligated with partially double stranded genome walker adapters. The adapter ligated restricted genomic DNA of 100 ng was used as template to PCR amplify the T-DNA integration site in the rice genome using genome walking specific primers listed in Supplementary Table 2. The amplified PCR product was cloned in TOPO vector and sequenced, and the sequencing results were subjected to BLAST analysis to identify the integration site in the rice genome.

### Expression Analysis of Transgenic Events

For northern analysis, the total RNA was isolated from 30 days old transgenic and *wt* plants, and rest of the procedure was completed according to Chandrasekhar et al. (2014). The total RNA (20 μg) from each rice line was fractionated by electrophoresis on 1% denaturing agarose gel containing formaldehyde, and the RNA samples were transferred onto a positive nylon membrane. The same RNA blot was hybridized with DIG labeled rice *EPSPS* probe (for both ‘S’ and ‘D’ transgenic lines) and *igrA* probe (for ‘D’ transgenic lines) consecutively in separate experiments. All further steps, including membrane washing, detection of northern signals, were completed according to the supplier’s instructions (Roche Diagnostics, Germany).

The semi-quantitative real time PCR (semi quantitative RT-PCR) was performed using primers EPSPS-F and EPSPS-R (Supplementary Table 2) to monitor the differential expression of native and transgene. The template cDNA of transgenic lines and *wt* plants were prepared using 2 μg total RNA with the help of SuperScript III (Invitrogen). For the expression analysis of *EPSPS* and *IgrA*, the PCR conditions were 94°C for 1 min, 55°C for 1 min, and 72°C for 1 min for 30 cycles with initial denaturation at 94°C for 4 min using gene specific primers. The expression of house-keeping rice actin 1 (*Act1*) gene was used as the internal standard in PCR.

### Confirmation of Introduced Mutation and *EPSPS* Transgene Expression

To differentiate between PCR amplified fragments from native and transgenic EPSPS transcripts and for confirmation of P173S mutation in transgenic lines, the column purified semi-quantitative real time PCR product of 200 ng (Supplementary Table 2) were digested with five units *Hind*III restriction enzyme. The unique *Hind*III restriction site present only in *OsmEPSPS* transgene helped to distinguish native and transgene *EPSPS* expression.

### Measurement of Growth and Physiological Parameters

For determining the level of tolerance of Swarna rice to glyphosate concentration at seed germination stage, the surface sterilized *wt* seeds were grown on half strength MS media containing different concentrations of glyphosate 10, 20, 30, 40, 50, and 100 μM for 20 days in glass jar bottles under growth chamber with a 12–12 light–dark cycle at 25°C. Further, to validate the effects of glyphosate on transgenic ‘S’ and ‘D’ lines, the surface sterilized seeds from transgenic lines (S1, S2, S3, D1, and D3) were grown on the half strength MS media supplemented with 100 μM of glyphosate. Seeds of *wt* were grown on same conditions with and without glyphosate served as positive and negative controls, respectively. The root and shoot lengths were measured on 20 days of seed germination and photographed.

The natural tolerance of Swarna rice against commercially available glyphosate marketed as Roundup (Isopropyl amine salt of glyphosate as active ingredient, 41.0% w/v) was determined by foliar spray applications of different doses of glyphosate (0.5, 1.0, 1.5, 2.0, 2.5, and 3.0 mL/L) on 30 days old *wt* plants under greenhouse conditions. Further to examine difference in the level of glyphosate tolerance by ‘S’ and ‘D’ plant groups, 1-month-old plants (8–12 leaf stage) from both ‘S’ and ‘D’ lines were uniformly foliar sprayed with glyphosate (2 mL/L) in green house condition at 26°C in day-light (12–12 h) conditions. Similar age group plants from *wt* were simultaneously foliar sprayed with water and with glyphosate (2 mL/L) which served as negative and positive controls, respectively, and handled alike. The glyphosate effect was regularly monitored carefully for the appearance of any visual symptoms and subsequently photographed.

### Statistical Analysis

The experimental data were reproduced and indicated in their respective figures, and pooled data were analyzed for one-way analysis of variance (ANOVA) followed by least significance difference (LSD) statistic at *p* ≤ 0.05 or 0.01 levels of significance.

## Results

### *In Silico* Analysis and Identification of P to S Glyphosate Resistant Mutation

Development of resistant weeds against herbicide is a global concern and has a great threat to modern agriculture. A total of 32 glyphosate-resistant weed biotypes has been identified in various parts of the world due to continuous and overuse of different herbicides. Weeds develop resistance against herbicide by changing amino acid sequence composition in the active site of targeted enzyme due to high herbicide selection pressure. To identify the common conserve proline position in the plant EPSPS polypeptides of different plant species, i.e., *Oryza sativa, Arabidopsis thaliana, Eleusine indica, Gossypium hirsutum, Nicotiana tabacum, Solanum lycopersicum, Sorghum halepense, Triticum aestivum, Lolium multiflorum*, and *Zea mays*, were sequence aligned. The analysis resulted in a consecutive 19 amino acid long conserved sequence group from 170 and 189 aa position (**Figure 1A**). The position of amino acid proline was found conserved in all the plant EPSPS protein and found at position 182 corresponding to *A. thaliana*, 180 to *L. multiflorum* and 173 aa in rice EPSPS.

Similarly, the sequence analysis of EPSPS from various glyphosate resistant and sensitive weed biotypes have shown single amino acid substitution at highly conserved P106 (P106S, P106T, P106A, and P106L) as shown in Supplementary Table 1 and **Figure 1A**. This single amino acid substitution endows moderate level of glyphosate resistance without any fitness cost on its enzyme kinetics. In a previous report, Chandrasekhar et al. (2014) have used the P173 mutation point and changed it to serine in rice EPSPS, and successfully developed glyphosate tolerant rice transgenic plants. From the sequence analysis and previous report, we identified the position of amino acid proline at 173 in rice EPSPS protein, and changed it with serine for developing glyphosate tolerant transgenic plants.

### Development and PCR Confirmation of Putative Transgenic Plants

**FIGURE 2:**
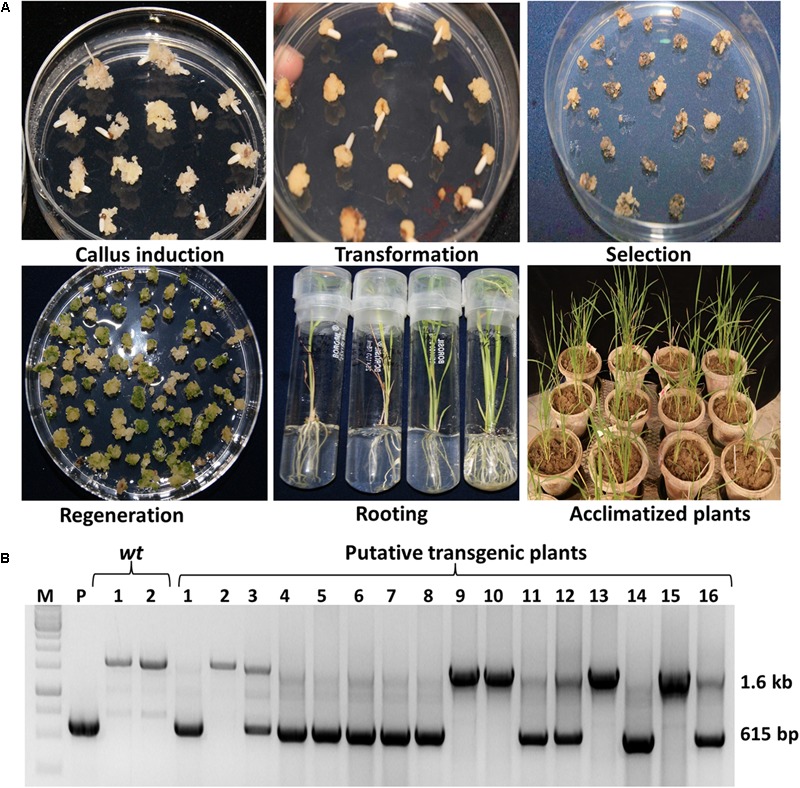
Generation of transgenic plants and their confirmation by PCR. Different stages of transgenic plant development following tissue culture **(A)**. PCR confirmation of putative transgenic plants using *EPSPS* gene specific internal forward and reverse primers. The upper amplified 1.6 kb band shows the presence of native *EPSPS* (with intron) gene while lower 615 bp PCR band confirms the presence of *OsmEPSPS* transgene (without intron) in both ‘S’ and ‘D’ transgenic groups **(B)**.

The *Agrobacterium* EHA105 strain carrying either single or double gene expression cassette was transferred separately into indica rice cultivar Swarna (**Figure 2A**). Following the rice tissue culture method, total 10 putative rice transgenic plants from ‘S’ and 6 from ‘D’ transgenic lines were generated (**Figure 2B**). All putative transgenic plants were initially screened for the integration of *OsmEPSPS* gene expression cassette using rice *EPSPS* gene specific internal screening primers EPSPS-F and EPSPS-R (Supplementary Table 2). The forward and reverse primers were designed from the coding regions of genomic *EPSPS* copy which only amplifies a DNA fragment of 1,677 bp in the *wt* plants that contain subsequent three introns (i.e., intron 1, 2, and 3). On the contrary, the *EPSPS* gene expression cassettes were made from the full length EPSPS coding sequence without introns, and hence the transgenic plants amplified a smaller DNA fragment of size 615 bp from the *OsmEPSPS* expression cassette along with 1,677 bp DNA fragment from the native *EPSPS* in PCR using above internal primers. The above PCR based strategy act as a marker to differentiate transgenic lines from the *wt* one. A total of 11 independent transgenic plants were found PCR positive for the integration of *OsmEPSPS* transgene in both ‘S’ and ‘D’ transgenic plants (**Figure 2B**). Out of 11 transgenic lines, seven transgenic plants were from ‘S’ group alone and remaining four plants were from ‘D’ group. The ‘D’ transgenic plants were further screened for the integration of *igrA* gene and a total three plants were seen PCR positive. We selected three best independent transgenic lines from each ‘S’ and ‘D’ transgenic group based on seed germination assay in the presence of 40 μM glyphosate (data is not shown) and these lines were used for further analysis.

### Molecular Confirmation and Transgenic Event Characterization

**FIGURE 3:**
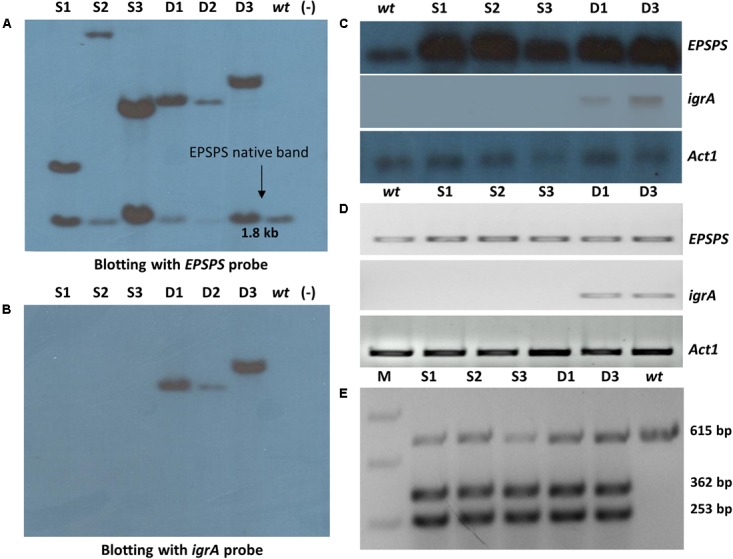
Molecular and expression analysis of transgenic plants. Southern blot signals confirm the presence of transgenes in ‘S’ and ‘D’ transgenic groups with *EPSPS* **(A)** and *igrA* gene probe **(B)**. Confirmation of *OsmEPSPS* and *igrA* transgenes expression by Northern blotting **(C)** and semi quantitative RT-PCR **(D)** in ‘S’ and ‘D’ transgenic lines. The rice *Act1* gene was used as reference gene in both experiments. Confirmation of P173S mutation in transgenic plants by restriction digestion of cDNA amplified PCR product of 615 bp with *Hind*III enzyme in *wt* and ‘S’ and ‘D’ group transgenic lines. The restriction digestion resulted in three bands of 615 bp, 362 bp and 253 bp, respectively, in all transgenic plants **(E)**. The digested and undigested products showed the presence of expression of both native *OsmEPSPS* and trans *OsEPSPS* genes, respectively.

The purified DNA of six PCR confirmed single and double gene transgenic plants along with *wt* rice plants was digested with *Nco*I restriction enzyme (which cuts the T-DNA before the probe region in *OsmEPSPS* expression cassette, and it is a non-cutter of *igrA* expression cassette) and initially probed with *EPSPS* gene (**Figure 3A**). All six transgenic lines showed two distinct southern positive signals, one signal around 1.8 kb region which was present in all transgenic lines including *wt* control, representing the native *EPSPS* copy. However, another signal represented the transgenic *OsmEPSPS* integration which appeared with distinct hybridization band pattern for each transgenic line. From the southern blotting, it was confirmed that all the transgenic lines from ‘S’ and ‘D’ groups showed single *OsmEPSPS* transgene integration. Furthermore, the transgenic lines D1 and D3 possessed same southern positive pattern which were considered as transgenic lines with same event. The same blot was reprobed with *igrA* gene. The transgenic plants from ‘D’ group showed clear single positive signals with different band pattern except for D1 and D3 lines (**Figure 3B**) which showed same hybridization pattern for both EPSPS and igrA probe, so considered as same transgenic events.

### Transgene Expression Analysis

The RNA blot analysis showed a single hybridization signal with EPSPS probe which was observed in all transgenic and *wt* plants (**Figure 3C**). However, the intensity of DIG-*EPSPS* signals were found high in all the transgenic lines from ‘S’ and ‘D’ groups compared to *wt* plant since both native and transgenic *OsmEPSPS* genes were expected to express simultaneously. Similarly, the blot was re-probed with DIG-labeled *igrA* probe, and a single distinct band signal appeared in D1 and D3 transgenic lines. Further, the *wt* plant and ‘S’ transgenic group did not show any signals with *igrA* probe (**Figure 3C**). The results confirm the active transcription of both the transgenes in transgenic rice plants. To ascertain the concentration of total RNA equally used in the present study, the blot was further reprobed with *Act1* gene which appeared with clear distinct positive signals having equal level of expression in all the plant samples (**Figure 3C**).

The expression of *OsmEPSPS* and *igrA* genes was further validated with the help of semi quantitative RT-PCR which showed a decreased expression of *EPSPS* gene in *wt* compared to transgenic lines from ‘S’ and ‘D’ groups (**Figure 3D**). Similarly, the transgenic lines from ‘D’ group showed an active expression of *igrA* gene. The expression of native *Act1* was used as internal reference gene showing equal expression in all plant samples (**Figure 3D**).

### Confirmation of Introduced Mutation in *EPSPS* Transgene

To distinguish the expression level of native *OsEPSPS* and transgenic *OsmEPSPS* in the ‘S’ and ‘D’ groups, we performed restriction–digestion analysis of amplified DNA products obtained from the respective cDNAs by reverse transcription of all transgenic and *wt* plants. The *Hind*III restriction enzyme could not digest 615 bp PCR product from *wt* sample while it was resulted in 253 bp and 362 bp DNA fragments in all transgenic plants along with the native 615 bp PCR band (**Figure 3E**). These results confirm the simultaneous expression of both native and *trans EPSPS* genes in all ‘S’ and ‘D’ transgenic rice lines. The introduction of unique *Hind*III restriction site, while replacing the existing proline with serine (P173S) in transgenic *OsmEPSPS* expression cassette, facilitated easy distinction between native and transgenic *EPSPS* gene transcripts.

### Effects of Glyphosate on Seed Germination

**FIGURE 4:**
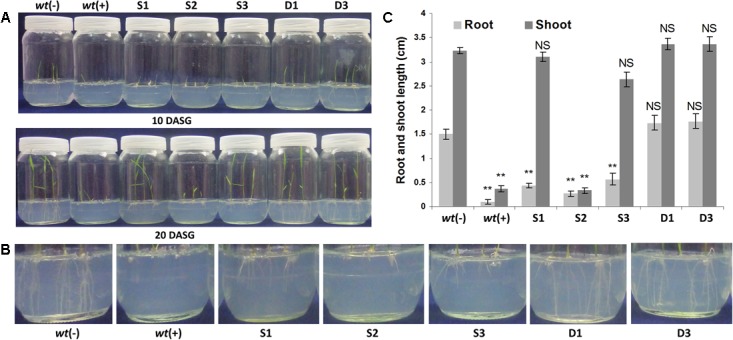
Seed germination study and morphological analysis of transgenic plants under glyphosate treatment. The T3 homozygous transgenic seeds were grown in the presence of 100 μM glyphosate. The seeds from ‘D’ group transgenic lines showed higher glyphosate tolerance, stayed green, and the root and shoot lengths were comparable with *wt* plants without glyphosate application **(A)**. However, the ‘S’ group transgenic lines showed poor root and shoot morphology and less tolerance to glyphosate **(B)**. *wt*(–) – wild type plants without glyphosate application; *wt*(+) – wild type plants with glyphosate application; S1, S2, and S3, ‘S’ transgenic lines; D1 and D3, ‘D’ transgenic lines; DASG, days after seed germination **(C)**.

The root growth inhibition, while germinating the *wt* rice seeds in different concentrations of glyphosate in MS medium, was started from 20 μM glyphosate concentration and there was a complete inhibition at and from 40 μM concentration onward. So, 40 μM concentration of glyphosate was regarded as the natural tolerance of Swarna rice at seed germination stage. To establish the performance of ‘S’ and ‘D’ transgenic plant lines in glyphosate, the sterilized rice seeds from ‘S,’ ‘D,’ and *wt* rice plants were grown on a half strength MS media supplemented with 100 μM of glyphosate [since the P173S mutation gives at least twofold to threefold resistance against glyphosate (Supplementary Table 1)], and the root and shoot growth was recorded after 20 days of seed inoculation. The seed germination assay showed that the transgenic rice seedlings expressing both *OsmEPSPS* and *igrA* genes, i.e., ‘D’ transgenic lines, had normal shoot and root growth in the presence of 100 μM of glyphosate which was comparable to *wt* rice seedlings without glyphosate application (**Figures 4A–C**). The ‘S’ group transgenic lines, expressing *OsmEPSPS*, exhibited a comparable shoot growth, however, the root growth was significantly inhibited (**Figures 4A–C**). These results clearly suggest that co-expression of both *OsmEPSPS* and *igrA* genes confers better glyphosate tolerance and phenology compared to ‘S’ transgenic plant lines expressing only *OsmEPSPS* gene. Further, we noticed that the transgenic lines did not show any phenological abnormalities or differences from *wt* plants.

### Physiological Performance of Transgenic Plants under Glyphosate Treatment

**FIGURE 5:**
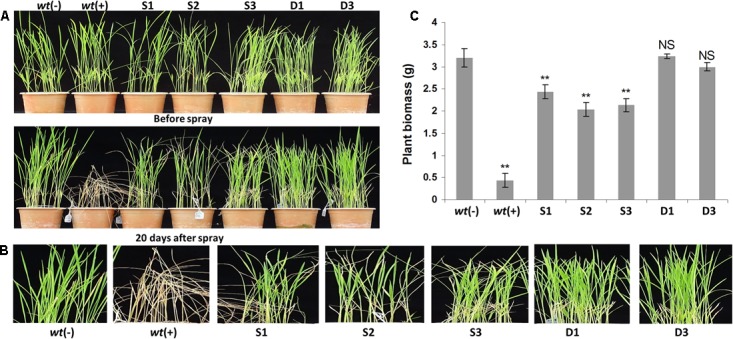
Physiological analysis of transgenic plants under foliar application of glyphosate. Morphology of 21 days old transgenic ‘S,’ ‘D,’ and *wt* plants upon foliar application of 3 mL/L Roundup herbicide **(A–C)**. The ‘D’ group transgenic lines showed higher glyphosate tolerance and possessed healthy and normal morphology comparable to *wt*(–) plants **(A–C)**. However, the transgenic lines from ‘S’ group showed a significant foliar injury and yellow fleshing **(A,B)**. *wt*(–) – wild type plants without glyphosate application; *wt*(+) – wild type plants with glyphosate application; S1, S2, and S3, ‘S’ transgenic lines; D1 and D3, ‘D’ transgenic lines.

**FIGURE 6:**
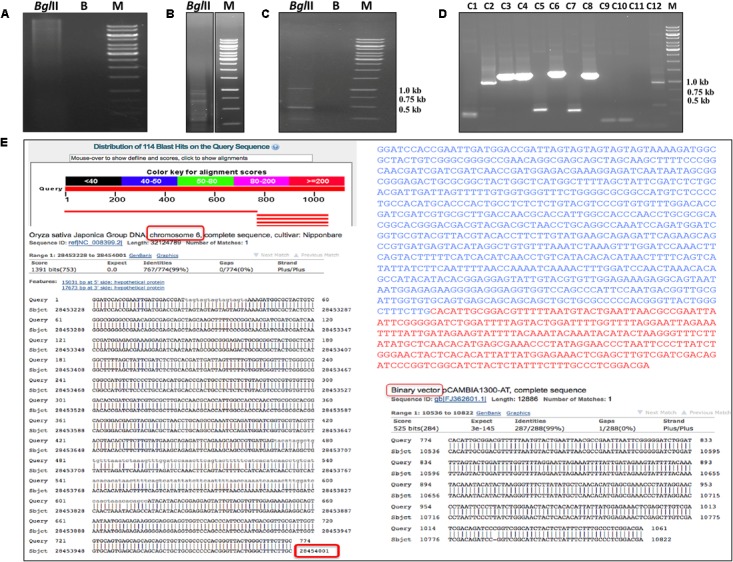
Identification of transgene integration by genome walk technique. Steps involved in genome walk technique. Restriction digestion of genomic DNA of ‘D3’ transgenic line (high glyphosate tolerant) with *Bgl*II enzyme **(A)**. Initial amplification of transgene integrated product using adaptor specific and T-DNA specific primers **(B)**. PCR amplification of transgene integrated product using first PCR product as template **(C)**. Confirmation of genome integrated T-DNA sequence cloned in TOPO vector by colony PCR **(D)**. Identification of transgene integration site in the rice genome using BLAST program **(E)**.

The ‘S’ and ‘D’ transgenic lines were screened by foliar spraying with 2 mL/L concentration of commercially used isopropyl amine salt of glyphosate herbicide (Roundup) and regularly monitored for any visual appearance of injury symptoms. The *wt* plants started showing typical yellow fleshing symptoms from the third day after glyphosate spray. Similarly, the ‘S’ group transgenic lines started showing visual symptoms from seventh day, however, we did not notice such symptoms or other necrotic fleshing in ‘D’ transgenic lines upon foliar spraying with glyphosate. Further, the negative *wt* plants completely died after 20 days of glyphosate spraying, while the ‘S’ transgenic plants showed high yellow fleshing and injuries in whole plant which were started with leaf tips, although these plants survived. However, the ‘D’ transgenic plants appeared healthy and there was no sign of any visual injury (**Figures 5A–C**), and the phenology was comparable to *wt* control plants without glyphosate treatment. We further identified the best transgenic event among ‘D’ transgenic lines (D1 and D3) by foliar treatment with next higher dose of 3 mL/L of glyphosate. The results showed that there were no symptoms of visible injury up to 7 days, and after that D1 transgenic lines started responding to the concentration earlier than D3 line. So, the D3 line was found best among all the glyphosate tolerant transgenic lines. Furthermore, the high glyphosate tolerant D3 transgenic line was subjected to genome walk to identify the transgene integration in the rice genome. The BLAST results confirmed that the T-DNA region was integrated in chromosome 6 at the nucleotide position 28454001 of the D3 transgenic line (**Figure 6**).

## Discussion

Glyphosate is one of the leading post-emergent, systemic, non-selective, and broad-spectrum herbicides with high unit activity and favorable toxicology. It indiscriminately kills all the plants by completely inhibiting EPSP synthase, a crucial enzyme of shikimate pathway in plants which is required for the biosynthesis of aromatic amino acids (tyrosine, phenylalanine, and tryptophan) and many other important plant metabolites. The plant EPSPS enzymes are highly sensitive to glyphosate and rapidly inhibited by its application. Enormous agricultural success has been achieved to develop commercially important glyphosate-tolerant transgenic crops using insensitive/tolerant EPSPS enzymes, e.g., corn, cotton, and soybean (Green and Owen, 2011). Although, these forms of EPSPS enzyme can tolerate higher amount of glyphosate application, the accumulated glyphosate molecules inside the plant cell start interfering with basic metabolic pathways, especially in photosynthesis processes, growing meristematic tissues and reproductive organs, resulting in leaf yellow fleshing and stunted growth of the plants along with low grain yield (Pline et al., 2002; Chen Y.C. et al., 2006; Liang et al., 2017). Moreover, the accumulated residual glyphosate molecules in the grains of transgenic crops are of much concern to human health (De Roos et al., 2005; George et al., 2010; Greim et al., 2015; Guyton et al., 2015).

In present study, the contribution of *igrA* gene to impart higher glyphosate tolerance was analyzed in the presence of glyphosate tolerant *OsmEPSPS* gene in rice transgenic plants. Previously, researchers have confirmed the glyphosate degrading efficiency of *igrA* gene alone in transgenic tobacco and rice plants (Vemanna et al., 2017). We developed two types of transgenic plants by overexpressing glyphosate tolerant P173S rice *OsmEPSPS* mutant gene alone and in combination with glyphosate degrading *igrA* gene. The transgenic lines of both groups, representing single and double glyphosate tolerance mechanisms, respectively, were compared for their glyphosate tolerance efficiencies with each other. The first transgenic rice lines were generated by overexpressing glyphosate tolerant P173S rice *OsmEPSPS* mutant gene without disturbing the native *EPSPS* locus. These transgenic lines showed normal phenotype and exhibited moderate level of glyphosate tolerance (100 μM) in germination study. Similar results have previously reported in many transgenic plants expressing proline to serine EPSPS mutation (Chandrasekhar et al., 2014).

The proline to serine substitution is one of the most common mutation point that has been identified in several glyphosate resistant weeds through natural selection, and confers glyphosate resistance without any detectable fitness cost (Supplementary Table 1). The enzyme kinetic properties of P173S EPSPS equivalent mutants from plants or bacteria exhibit a higher *K*_m_ value for its natural substrate PEP (phosphoenolpyruvate) without any effect on *V*_max_ of the reaction, with a significant decrease in glyphosate binding affinity (Funke et al., 2009; Pollegioni et al., 2011). The multiple findings of similar P to S mutation, reported in various glyphosate tolerant weeds in different parts of the world and in different time periods, confirm this mutation point as a universal hot spot for glyphosate resistance without losing EPSPS activity (Baerson et al., 2002). The directed evolution strategies have also been used to generate glyphosate tolerant EPSPS mutants, but these mutants often display undesirable enzyme kinetics. These mutations reduce catalytic efficiency of EPSPS enzyme due to decrease in binding affinity for substrate PEP that results in decrease in overall fitness and survival of the plant (Bradshaw et al., 1997; for review, see Pline-srnic, 2006). Recently, it was reported that the naturally occurring EPSPS double amino acid substitution tyrosine to isoleucine and proline to serine (referred as TIPS) showed higher tolerance to glyphosate in goosegrass. However, the TIPS glyphosate tolerant mutation resulted in fitness disadvantages compared to its wild type control (Yu et al., 2015).

Several glyphosate-insensitive type II *EPSPS* genes and glyphosate tolerant *EPSPS* mutants (generated through directional evolution strategies) from various sources have been used for the development of glyphosate tolerant transgenic plants (Pline-srnic, 2005, 2006; Green, 2009). However, the accumulated glyphosate residue inside the plant cell is a major concern which severely affect the actively growing meristematic tissues and results in overall fitness cost and low grain yield. To overcome this problem, an additional glyphosate detoxifying (degrading) enzyme along with glyphosate insensitive/tolerant *EPSPS* gene is used which results in development of more robust glyphosate tolerant transgenic plants. Many glyphosate metabolizing enzymes like *GAT*, *GOX*, *GO*, and *DAAO* have been characterized from lower group organisms, and used in developing glyphosate tolerant transgenic plants. The selective variants of *GAT* genes from various sources were used in transgenic maize (Castle et al., 2004), soybean (Delaney et al., 2008), and tobacco (Dun et al., 2014; Liu et al., 2015) either alone or in combination with glyphosate insensitive/tolerant *EPSPS* genes. Similarly, improved version of *GOX* gene from *Ochrobactrum anthropic* and CP4 *EPSPS* from *Agrobacterium* were used to develop glyphosate tolerant transgenic wheat (Zhou et al., 1995) and transgenic maize (Howe et al., 2002). The codon optimized variant of *GO* from *Bacillus subtilis* was used to develop glyphosate tolerant transgenic alfalfa plants (Nicolia et al., 2014). Recently, Han and their team developed glyphosate tolerant *Arabidopsis thaliana* plants by over-expressing *DAAO* gene from *Bradyrhizobium japonicum* (Han et al., 2015).

The second type of glyphosate tolerant transgenic rice plant lines were developed by co-expressing glyphosate degrading encoding *igrA* gene and glyphosate tolerant P173S mutant rice *OsmEPSPS* gene which showed a higher glyphosate tolerance over the *OsmEPSPS* overexpression transgenic lines, as confirmed by various expression and physiological experiments. Recently, the *igrA* gene has alone been used for development of glyphosate tolerant transgenic tobacco and rice plants and validated its role in effective detoxification of glyphosate in transgenic plants (Vemanna et al., 2017). The higher level of glyphosate tolerance in rice both gene co-expression transgenic lines can possibly be explained by the continuous degradation of accumulated glyphosate by igrA enzyme, and by the significant decrease in glyphosate interaction with OsmEPSPS. Both the independent strategies operated together to confer higher glyphosate tolerance.

Pyramiding of both glyphosate tolerance and degradation strategies, and simultaneous overexpression of both physically linked genes provided the synergistic effects for higher glyphosate tolerance. As expected, the transgenic rice seedlings expressing both *igrA* and *OsmEPSPS* genes showed better growth, longer roots and shoots, and higher biomass compared to the *OsmEPSPS* rice transgenic lines in presence of glyphosate (**Figure 4**). We have also reported the similar results when glyphosate (2 mL/L) was sprayed on both the transgenic lines (**Figure 5**). The co-expression of *igrA* and P173S mutant *OsmEPSPS* resulted in higher tolerance compared to P173S mutant *OsmEPSPS* alone. Glyphosate is a potent inhibitor of EPSPS enzyme even in micromolar concentration and the enzyme kinetics of igrA is slow to degrade the glyphosate which subsequently results in low glyphosate tolerance when expressed alone (Vemanna et al., 2017). However, the sustained activity of igrA can reduce the ‘true’ intra cellular concentration of glyphosate to inhibit the EPSPS activity. The EPSPS enzyme is a part of shikimate pathway that is operated inside the chloroplast, while the supporting igrA protein in the present study was expressed constitutively in the cytoplasm. Further studies are required to evaluate the role of igrA in effective detoxification of intra cellular glyphosate either by targeted expression in chloroplast to support EPSPS enzyme or by tissue specific expression in phloem tissues to prevent the active translocation and selective accumulation of glyphosate in meristematic tissues that arrests plant growth and development.

## Conclusion

The transgenic plants co-expressing *OsmEPSPS* and *igrA*, having both glyphosate tolerance as well as degradation mechanisms, showed enhanced glyphosate tolerance compared to transgenic plants expressing only *OsmEPSPS* gene. The study pointed out the importance of igrA as a supportive mechanism in imparting glyphosate tolerance along with the use of glyphosate tolerant EPSPS enzyme for the development of glyphosate tolerant crops for effective weed management. The study also suggests a significant role of *igrA* gene in transgenic plants to degrade the accumulated glyphosate in actively growing meristematic tissues.

## Author Contributions

DF and MR conceived the idea and designed all the experiments. DF, AA, DJ, and BB were involved in generation of transgenics and completed molecular analysis. DF, BR, VS, and MM helped in physiological analysis of transgenic plants. DF, RY, and PV assisted in molecular analysis. DF, VA, and MR critically analyzed the data and wrote the manuscript.

## Conflict of Interest Statement

The authors declare that the research was conducted in the absence of any commercial or financial relationships that could be construed as a potential conflict of interest.
