# Supplementary material for: Co-expression of P173S Mutant Rice EPSPS and igrA Genes Results in Higher Glyphosate Tolerance in Transgenic Rice
Source: Front Plant Sci. 2018 Feb 13;9:144. doi: 10.3389/fpls.2018.00144 (PMC5816812; doi:10.3389/fpls.2018.00144)
Supplement: Supplementary file 1 [file Table_1.DOC]

Table SI 1. Glyphosate resistant weeds with P106 mutations reported around the world.

| **Name of the weed plant** | **Common name** | **Change in amino acid** | **Reference** |
| --- | --- | --- | --- |
| *Eleusine indica* | Goosegrass | Pro106Ser | (Kaundun et al., 2011) |
| *Lolium perenne (multiflorum)* | Italian Ryegrass | Pro106Ser | (Perez-Jones et al., 2007) |
| *Lolium rigidum* | Rigid Ryegrass | Pro106Ser | (Simarmata and Penner, 2008) |
| *Amaranthus tuberculatus* | Tall Waterhemp | Pro106Ser | (Nandula et al., 2013) |
| *Echinochloa colona* | Junglerice | Pro106Ser | (Alarcón-Reverte et al., 2013) |
| *Eleusine indica* | Goosegrass | Pro106Thr | (Ng et al., 2004) |
| *Lolium rigidum* | Rigid Ryegrass | Pro106Thr | (Wakelin and Preston, 2006) |
| *Lolium rigidum* | Rigid Ryegrass | Pro106Ala | (Yu et al., 2007) |
| *Lolium perenne (multiflorum)* | Italian Ryegrass | Pro106Ala | (Jasieniuk et al., 2008) |
| *Lolium rigidum* | Rigid Ryegrass | Pro106Leu | (Kaundun et al., 2011) |
| *Digitaria insularis* | Sourgrass | Pro106Thr | (De Carvalho et al., 2012) |
| *Echinochloa colona* | Junglerice | Pro106Thr | (Alarcón-Reverte et al., 2015) |
| *Echinochloa colona* | Junglerice | Pro106Leu | (Han et al., 2016) |

References

Alarcón-Reverte, R., García, A., Urzúa, J., and Fischer, A. J. (2013). Resistance to Glyphosate in Junglerice (Echinochloa colona) from California. *Weed Sci.* 61, 48–54. doi:10.1614/WS-D-12-00073.1.

Alarcón-Reverte, R., García, A., Watson, S. B., Abdallah, I., Sabaté, S., Hernández, M. J., et al. (2015). Concerted action of target-site mutations and high EPSPS activity in glyphosate-resistant junglerice (Echinochloa colona) from California. *Pest Manag. Sci.* 71, 996–1007. doi:10.1002/ps.3878.

De Carvalho, L. B., Alves, P. L. D. C. A., González-Torralva, F., Cruz-Hipolito, H. E., Rojano-Delgado, A. M., De Prado, R., et al. (2012). Pool of resistance mechanisms to glyphosate in digitaria insularis. *J. Agric. Food Chem.* 60, 615–622. doi:10.1021/jf204089d.

Han, H., Yu, Q., Widderick, M. J., and Powles, S. B. (2016). Target-site EPSPS Pro-106 mutations: Sufficient to endow glyphosate resistance in polyploid Echinochloa colona? *Pest Manag. Sci.* 72, 264–271. doi:10.1002/ps.4038.

Jasieniuk, M., Ahmad, R., Sherwood, A. M., Firestone, J. L., Perez-Jones, A., Lanini, W. T., et al. (2008). Glyphosate-Resistant Italian Ryegrass (Lolium multiflorum) in California: Distribution, Response to Glyphosate, and Molecular Evidence for an Altered Target Enzyme. *Weed Sci.* 56, 496–502. doi:10.1614/WS-08-020.1.

Kaundun, S. S., Dale, R. P., Zelaya, I. A., Dinelli, G., Marotti, I., McIndoe, E., et al. (2011). A novel P106L mutation in EPSPS and an unknown mechanism(s) act additively to confer resistance to glyphosate in a South African lolium rigidum population. *J. Agric. Food Chem.* 59, 3227–3233. doi:10.1021/jf104934j.

Nandula, V. K., Ray, J. D., Ribeiro, D. N., Pan, Z., and Reddy, K. N. (2013). Glyphosate Resistance in Tall Waterhemp ( Amaranthus tuberculatus ) from Mississippi is due to both Altered Target-Site and Nontarget-Site Mechanisms. *Weed Sci.* 61, 374–383. doi:10.1614/WS-D-12-00155.1.

Ng, C. H., Wickneswary, R., Salmijah, S., Teng, Y. T., and Ismail, B. S. (2004). Glyphosate resistance in Eleusine indica (L.) Gaertn. from different origins and polymerase chain reaction amplification of specific alleles. *Aust. J. Agric. Res.* 55, 407–414. doi:10.1071/AR03155.

Perez-Jones, A., Park, K. W., Polge, N., Colquhoun, J., and Mallory-Smith, C. A. (2007). Investigating the mechanisms of glyphosate resistance in Lolium multiflorum. *Planta* 226, 395–404. doi:10.1007/s00425-007-0490-6.

Simarmata, M., and Penner, D. (2008). The Basis for Glyphosate Resistance in Rigid Ryegrass (Lolium Rigidum) from California. *Weed Sci.* 56, 181–188. doi:10.1614/WS-07-057.1.

Wakelin, A. M., and Preston, C. (2006). A target-site mutation is present in a glyphosate-resistant Lolium rigidum population. *Weed Res.* 46, 432–440. doi:10.1111/j.1365-3180.2006.00527.x.

Yu, Q., Cairns, A., and Powles, S. (2007). Glyphosate, paraquat and ACCase multiple herbicide resistance evolved in a Lolium rigidum biotype. *Planta* 225, 499–513. doi:10.1007/s00425-006-0364-3.
