# Supplementary material for: Co-expression of P173S Mutant Rice EPSPS and igrA Genes Results in Higher Glyphosate Tolerance in Transgenic Rice
Source: Front Plant Sci. 2018 Feb 13;9:144. doi: 10.3389/fpls.2018.00144 (PMC5816812; doi:10.3389/fpls.2018.00144)
Supplement: Supplementary file 2 [file Table_2.DOC]

Table SI2: List of the primers used in study

| **S. No.** | **Primer name** | **Sequence** |
| --- | --- | --- |
| **1.**  **2.**  **3.**  **4.**  **5.**  **6.**  **7.**  **8**  **9.**  **10.**  **11.**  **12.**  **13.**  **14.**  **15.**  **16.**  **17.**  **18.**  **19.**  **20.**  **21.**  **22.** | EPSPS A F  EPSPS A R  EPSPS B F  EPSPS B R  EPSPS-F  EPSPS-R  hpt F  hpt R  W-1  W-2  Specific primer (SP-1)  Specific primer (SP-2)  EPSPS-PF  EPSPS-PR  EPSPS-TF  EPSPS-TR  Act2-PF  Act2-PR  Act2-TF  Act2-TR  IgrA-F  IgrA-R | 5’-ATGGCGGCGACCATGGCGTC-3’  5’-TGTCAAGCTTCGCATTGCAGTTCCAG-3’  5’-ATGCGAAGCTTGACAGCAGCCGTGACT-3’  5’-TCAGTTCCTGACGAAAGTGC-3’  5’- GTCCAAGTCGCTCTCCAACA -3’  5’- TGCTCTGCCTTCACACCAAA -3’  5’- GCCTGAACTCACCGCGACG -3’  5’- CAGCCATCGGTCCAGACG -3’  5’-CTAATACGACTCACTATAGGG-3’  5’-GGGCGGCCGCCCGGGCGATC-3’  5’- ATTTCGATGATGCAGCTTGGG-3’  5’- CGTCCGAGGGCAAAGAAATAG - 3’  5’- ACGTAGGCAGTACACGTAGAC - 3’  5’- GGTCGCCGCCATTGCCGGCGG - 3’  5’ - ACTGAGCTTTTAAAAGAGTG - 3’  5’ - AGAAGGCCAAATTATTCTT - 3’  5’ - TTTGATTCCTTGAGTTTACG - 3’  5’ - CATTTATTTTAACTGATCTG - 3’  5’ - ATGTGGCCTAGCTGTATCTT - 3’  5’ - TCAAGAATCTCAAATCAATT - 3’  5’ - ATGCACCGCGAGGACGACTC - 3’  5’ - ACTAGTAGTCGAGGGCGACT - 3’ |
